# Vitamin D and hip protectors in osteosarcopenia: a combined hip fracture preventing approach

**DOI:** 10.1007/s11154-024-09907-8

**Published:** 2024-10-01

**Authors:** Alessandro Giustina, Andrea Giustina

**Affiliations:** 1 Department of Aerospace Engineering, Politecnico Di Milano, Via La Masa 34, Milan, 20156 Italy; 2 Institute of Endocrine and Metabolic Sciences, San Raffaele Vita-Salute University and IRCCS San Raffaele Hospital, Milan, Via Olgettina 60, 20132 Italy

**Keywords:** Osteoporosis, Hip fracture, Falls, Hip protectors, Prevention strategies, Sarcopenia, Osteosarcopenia, Vitamin D

## Abstract

Osteosarcopenia is an emerging clinical condition highly prevalent in the older people. Affected subjects due to their intrinsic skeletal fragility and propensity to falls are at elevated risk of hip fractures which can increase morbidity and mortality. Strategies for attenuating the impact of predisposing factors on hip fractures are not yet well defined and should derive from multidisciplinary care and collaborations. Our aim was to narratively review available data on the preventive role of vitamin D and hip protectors on hip fractures in older patients with sarcopenia. Older subjects are at high risk of vitamin D deficiency and of falls due to several concomitant factors besides osteosarcopenia**.** Vitamin D protective actions against hip fractures may be mediated by both skeletal (increased mineralization) and extra-skeletal (reduced risk of falls) actions. Hip protectors may act downstream attenuating the effects of falls although their use is still not yet enough widespread due to the suboptimal compliance obtained by traditional hard devices. Concomitant use of vitamin D and hip protectors may represent an effective strategy in the prevention of hip fractures which need to be tested in ad hoc designed clinical trials.

## Introduction

Hip fracture is the most devastating type of osteoporosis-related fracture, and is a major worldwide public health problem with a high socioeconomic burden together with elevated morbidity and mortality rate [1]. Thus, it is crucial to understand the risk and protective factors to create an effective hip fracture prevention strategy [2].

The two key predisposing factors to hip fractures are osteoporosis/osteopenia [3] and falls [4]. Interestingly, sarcopenia which is particularly frequent in the older people [5], may be on one hand often associated with osteoporosis (condition modernly defined osteosarcopenia which possibly also associated with obesity is an emerging geriatric syndrome) [6, 7] and on the other hand may predispose to an increased risk of falls [8].

In addition to a brief review of epidemiology and different risk factors of hip fractures, we will in this article discuss specifically role of osteosarcopenia and falls as main causes of hip fractures and role of vitamin D for their prevention as protective factor against fracturative events.

Our review will also focus on the state of the art as well as the perspectives for the use of hip protectors in the mitigation of the clinical consequences of falls and consequently their contribution to the reduction of the risk of hip fractures in synergy with vitamin D supplementation in osteosarcopenia.

## Hip fractures

### Epidemiology

Data collected from 2014 to 2015 from the Korean registry showed that femoral neck and intertrochanteric fractures accounted for more than 95% of all femoral fractures [9] as previously reported [10]. The global burden of hip fracture was shown to remain high by a collection of data of the last 30 years involving more than 200 Countries from 1990 to 2019 with a relevant increase in incidence among older adults. Besides age and falls, also female gender was associated with higher incidence and prevalence of hip fractures as compared to males, although the male to female ratio of the incidence increased over time (from 0.577 in 1990 to 0.612 in 2019) [11], indicating possible risk of underestimation and ineffective prevention, particularly in males [11], who, in another study collecting data from patients hospitalized with hip fracture in 19 countries, used less anti-osteoporosis drugs than females, had increased rates of all-cause mortality, and an excess projected hip fracture risk in the next two decades [12]. Interestingly, in the same international study, few patients were started on treatment in the year after hip fracture treatment (from a median of 11.5% in Germany to 50.3% in the UK) [12].

### Risk factors

Hip fractures are in most instances direct consequence of falls. Therefore, all conditions predisposing to falls both intrinsic to the patient (such as multimorbidity, chronic treatment with hypnotics and tricyclic antidepressants, and sarcopenia) and environmental are obvious risk factors for hip fractures [13]. In fact, in a recent retrospective study, 3 year outpatient data from nearly 8000 community dwelling older adults (predominantly females) were collected. In multivariate analysis adjusted for age and sex multimorbidity was found to be associated with higher risk of hip fracture (OR 1.12) and each episode of fall increased risk of hip fracture by about 1.7-folds [14]. The fall risk may also be related to the mass of the psoas and spine extensor muscle and their weakness may lead to an increased risk of falls and of different hip fracture [15]. Inasmuch, poor vision may also be a relevant and independent predicting factor for hip fractures [16].

Other well recognized risk factors for hip fractures are personal or family history of fractures and post-menopausal, age-related or glucocorticoid induced and other forms of secondary and drug induced osteoporosis [17–21], low body mass index, institutionalization, oncologic conditions or advanced stages of chronic diseases such as obstructive pulmonary disease [22], liver and renal insufficiency, cardiovascular and neurologic diseases, diabetes mellitus and malabsorptive conditions may be related to an increased risk of fractures [23–25].

Interestingly, most of these above mentioned conditions are burdened by high prevalence of hypovitaminosis D [26–29]. In fact, vitamin D may have protective effects against hip fractures due to its both skeletal and extraskeletal pleiotropic effects [30, 31] since on one hand, it acts improving calcium absorption and bone mineralization [32, 33] and on the other hand it may improve muscle quality and performance thus contributing to decreased risk of falls [34, 35] with what we can modernly define “anti-osteosarcopenic” effect.

Several hip geometry features have been reported to be related to hip fracture risk including but not limited to length of the hip axis and to the femoral neck axial length and width, femoral shaft diameter and cortical thickness [36]. Differences in proximal femur geometry lead to different types of hip fracture [37]. Moreover, bone strength is negatively impacted by mechanical unloading as it was seen in patients undergoing prolonged lock-down during the COVID-19 pandemic [38, 39]. In fact, bone microstructural changes are impacted by neuromuscular function as seen in patients with reduced appendicular lean mass who experienced accelerated cortical bone loss and increased risk of falls and hip fracture [40].

Finally, lack of use of external protections [41] may be an adjunctive risk factor for hip fractures in a patient with osteosarcopenia undergoing falls. In the next paragraphs, we will review the most recent pathophysiological aspects of osteosarcopenia and the role of vitamin D and hip protectors in the prevention of hip fractures.

### Clinical consequences

#### Morbidity

Post-fracture 5 year quality of life data on more than 35,000 hip fractures (median age 83 years; more than 60% females) from the Norwegian Hip Fracture Registry were collected [42]. Quality of life investigated with EQ-5D-3L scoring tool decreased from 0.81 (pre-fracture) to 0.66 (4 months after fracture) and then remained stable for next 36 months and highly impaired as compared to data from the Norwegian Patient Registry and Statistics Norway. Decrease in quality of life appeared to be more pronounced in males of advanced age, living in care facilities, with severe comorbidity, cognitive impairment and lower levels of income and education, [43].

Moreover, mobility is often impaired after hip fractures and less than half of hip fractured patients are able to return to their pre-fracture mobility conditions in close relationship with general health status and quality of life [44].

#### Mortality

Large majority of hip fractured patients undergo surgical management [45]. In a retrospective study including more than 94.000 patients only 3.2% of them were not operated [46]. Non-operative management was burdened by high mortality rates (37.6% at 7 days and 57.1 at 30 days) and was related to advanced age, institutionalization, lack of independence in activities of daily living and mobility [46], ADL score [47] and dementia [48].

However, mortality after hip fractures is still high even when the patient is managed surgically [49]. In fact, in the above mentioned recent 4 year retrospective Chinese study including more than 3000 patients above the age of 50 with intertrochanteric fracture, overall mortality in patients managed surgically was 1.57% at 3 months increasing to 12% at 36 months exceeding mortality rates of the general population. Renal insufficiency, metastatic tumors, hypoproteinemia and age were related to increased mortality risk [46].

A recent meta-analyis including more than 462,000 patients from 33 studies reported also as possible strong predictive factors for early (30 days) post-surgical mortality age, male sex, institutionalization, and metastatic tumors. Additional weaker predictors were chronic kidney and heart failure, dementia, diabetes mellitus, anemia and oncologic history [50].

Finally, in another recent meta-analysis including more than 4400 patients from 9 studies, vitamin D insufficiency did significantly associate with increased mortality at 1 and 2-year follow-up (OR 1.37 and 1.78 respectively)) whereas severe vitamin D deficiency more than doubled mortality risk (OR 2.08) although after adjustment for possible confounders, observed increase in the rate of mortality rate did not maintain statistical significance [51].

## Osteosarcopenia

### Pathophysiology and epidemiology

Osteosarcopenia is the combination of decreased bone density as defined by WHO [52] and low muscle mass/strength leading to impaired physical function (sarcopenia) based on more controversial criteria proposed by different international organizations [53, 54]. The association of these two conditions recognizes unique pathophysiological mechanisms and risk factors. In fact, besides aging and frailty or poor general status, physical activity and nutrition [55] several hormone deficiencies including lack of vitamin D may play a crucial role in the pathogenesis of osteosarcopenia [56, 57]. Specifically, the growth hormone (GH)/insulin-like growth factor-1 (IGF-1) axis is a key regulator of bone and muscle metabolism [58, 59]. As a consequence reduced or excessive levels of GH and IGF-1 may be associated with an increased risk of fracture [60–63] and altered body composition [64, 65]. Moreover, aging-related or glucocorticoid-related GH deficiency may possibly lead to occurrence of worsening of osteosarcopenia [66–68].

Depending on the definition (which is mainly influenced by the criteria used for assessment of sarcopenia) [53] the prevalence of osteosarcopenia may be variable based on sex ranging from 12 to 64.3% in women and from 4 to 59.4% in men. Higher prevalence of osteosarcopenia in women may be due to combined lower muscle and bone parameters vs men of similar age, particularly after the age of menopause [55].

### Clinical consequences

Clinical consequences of osteosarcopenia include falls, fractures, reduced quality of life and increased risk for hospitalization and mortality [69]. Moreover, several studies have shown that osteosarcopenia was also associated with worsened ADL in older adults [70].

In a recent meta-analysis, although based prevalently on cross-sectional data and focused on specific subpopulations, osteosarcopenia was reported to be significantly correlated with increased risk of fracture and to a lesser degree with falls as compared to non osteosarcopenic subjects [71]. However, no consensus was reached among different studies about a possible incremental fracture risk in osteosarcopenic as compared to only osteoporotic males.

Sexual dimorphism of osteosarcopenia-related fracture risk may be due to the potent pro-sarcopenic effect of the decrease in testosterone levels in the aging man [72] as well as to the wide use of anti-osteoporotic drugs in females which, at least in part, may attenuate the pro-osteoporotic effect of post-menopausal estrogen loss [73]. A similar concept may apply to vitamin D deficiency since women are also more frequently supplemented with vitamin D and as a consequence males are at increased risk of hypovitaminosis D than women [74–77].

## Vitamin D in the prevention of hip fractures

### Physiology of vitamin D

Vitamin D is a pleiotropic hormone prevalently synthesized in the skin as cholecalciferol after exposure to sunlight and to a far lesser extent introduced with food unless it is fortified with vitamin D [31]. Therefore, subjects with insufficient exposure to sun radiations, such as most of the institutionalized older people, almost invariably have a poor vitamin D status which, due to ubiquitous vitamin D receptor expression, can have serious detrimental impact on several skeletal and extraskeletal endpoints [30]. In fact, vitamin D and calcium are key players in bone mineralization which is impaired when vitamin D levels are not sufficient since in this case intestinal calcium absorption is impaired [30, 31].

### Effect on bone loss

#### Vitamin D deficiency

Reduced BMD (in the osteoporotic or even osteopenic range) is associated with increased fracture risk [78, 79] although in recent years also impaired quality of bone has been proposed as a key determinant of risk of fracture particularly in patients with endocrine-related or other secondary forms of skeletal diseases [80–84] who, in fact, may undergo specifically vertebral fractures in the presence of normal BMD [85–88]. Inasmuch, often in older subjects, who are very frequently vitamin D deficient, secondary hyperparathyroidism may be linked to impaired BMD and increased fracture risk [89, 90]. In fact, when vitamin D levels are mildly reduced there is a trend towards serum calcium reduction which causes a compensatory PTH rise [89]. This, in turn, on one hand restores normal serum levels of calcium through its increased absorption from the GI tract by improving conversion of inactive to active vitamin D [31]. However, on the other hand, this compensatory hyperparathyroidism increases bone turnover and cortical bone resorption [30, 89].

Moreover, in case of persistent severe hypovitaminosis D older subjects may develop osteoporomalacia, i.e. a condition in which reduced BMD is associated with reduced mineralization of the newly produced osteoid tissue [89]. In fact, in clinical trials as well as in clinical practice treatment with anti-osteoporotic and particularly anti-resorptive drugs should be combined with vitamin D supplementation, generally in form of cholecalciferol, is order to maximize their anti fracture action [91].

Therefore, vitamin D deficiency may be linked not only to decreased BMD but also to an increased fracture risk in the older people [89] with an estimated 5–10% contribution to the global burden of incident hip fractures [89]. In fact, either statistically significant negative relationship between serum 25 (OH) vitamin D levels and risk of fracture or significantly decrease in risk of hip fracture risk with 25(OH)D levels above 62.5 nmol/L were reported [92].

#### Vitamin D supplementation

Statistically significant decreased hip fracture risk (from 16 to 39% in 8/13 meta-analyses) with vitamin D + calcium was found in an umbrella review including meta-analyses of RCTs on vitamin D supplementation. These findings were impacted at least in part by data on fractures occurring in institutionalized and frail older individuals [93].

In most of these studies, baseline 25(OH)D levels in the blood were very low (< 10 ng/ml) as it was the calcium intake, and daily 800–1000 IU doses of cholecalciferol were prevalently used [94] whereas bolus high doses over 50,000 IU monthly of vitamin D are not anymore recommended since they may paradoxically increase fall risk [95].

Unfortunately, whereas data on the negative impact on bone of low circulating vitamin D levels although deriving prevalently from observational or retrospective poorly controlled studies are quite consistent across the literature, much less is the concordance between observational and retrospective studies, meta-analysis and randomized controlled trials (RCTs) concerning the effect on bone of vitamin D supplementation. In fact, observational and retrospective studies, although providing valuable insights, may be, at least theoretically less informative than RCTs since affected by potential confounders [96] and biases including age of study subjects, duration of follow-up, vitamin D dose and prevalence of hypovitaminosis D and comorbidities such as obesity [97, 98].

In fact, recently published RCTs did not report statistically significant protective skeletal action of vitamin D supplementation [99]. However, they were also affected by methodological problems which made difficult their interpretation since they enrolled predominantly vitamin D sufficient middle-aged subjects undergoing variable vitamin D supplementation schedules using in some cases quite elevated vitamin D doses [100]. Interestingly, Chiloiro et al. in a retrospective non randomized study recently shown that cholecalciferol supplementation decreased incidence of morphometric vertebral fractures in acromegaly [101].

### Effect on falls

Falls are most direct cause of fractures of the hip in older people. In fact, it is estimated that approximatively one third of persons older than 65 years of age may fall each year and more than three quarters of fractures may be the consequence of falls [102]. Hence, fall prevention is mandatory in order to reduce incident hip fractures [103]. This may be obtained removing environmental factors predisposing to falls (eg carpets, irregular floors and shoes with high heels). Physical and individual factors such as orientation (and personal history) of falls can not easily be prevented or anticipated [104, 105].

#### Vitamin D deficiency

The most relevant factors predicting the risk of falls include muscle weakness and gait or balance deficits which can be associated with vitamin D deficiency particularly in older subjects [106]. In fact, selective type 2 muscle fibers, decreased vitamin D receptors expression and altered balance can be linked to hypovitaminosis D [107, 108].

In fact, in a recent Brazilian cross-sectional study including more than 200 community dwelling individuals over 80 years prevalently vitamin D deficient (25(OH)D < 20 ng/mL) blood levels of 25(OH)D resulted positively and significantly associated with the scores of the functional reach test (*p* = 0.037) a test consisting of 10 activities which explore different aspects of balance [109].

Moreover, an intra trial evaluation of 25(OH)D levels in more than 400 over 65 years of age participants in the Boston STOP IT trial showed that mean 25(OH)D levels were significantly associated with fall risk according to a U-shaped curve and that 25(OH)D range associated with the lowest fall risk was from 20 to 40 ng/mL [110].

#### Vitamin D supplementation

A recent meta-analysis on 29 randomized placebo-controlled interventional trials showed that muscle strength was ameliorated by vitamin D administration although apparently muscle mass was not improved likely at least in part since few specific studies were included [111].

There is quite convincing evidence that vitamin D supplementation may reduce the fall risk although Literature is not fully concordant on this outcome [34] and, as mentioned above for skeletal endpoints, the quality and relevance of the evidence is not always high with possible over-reliance on observational and retrospective studies.

In fact, more than a decade ago a meta-analysis suggested that a dose of vitamin D around 800 IU was able to reduce fall risk by about 15% [112]. However, this dose of vitamin D was not able to reduce the risk of falls in post-menopausal or older women [113, 114]. On the other hand, a relatively small intervention trial investigating the effect of different vitamin D doses reported significantly lower risk of falls in postmenopausal women taking 1,600—3,200 IU of vitamin D per day [115]. Moreover, the ViDA trial using 100,000 IU of vitamin D per month did not report a preventive effect on falls in more than 5,000 individuals followed for approximatively 3 years [116].

Interestingly, vitamin D status before starting supplementation may be a key factor in predicting the outcome of vitamin D administration on falls as on other clinical outcomes since being vitamin D a threshold nutrient, beneficial outcomes can be expected only if study populations are vitamin D deficient [117]. In fact, in the above mentioned mega trial majority of enrolled participants were either vitamin D sufficient or mildly insufficient [116]. In this regard, another meta-analysis of trials conducted on vitamin D supplementation and fall risk showed that only in studies enrolling patients with low 25(OH)D values at baseline a protective effect was seen [118]. Moreover, a recent meta-analysis of 32 studies testing supplementation with daily doses of 800 to 1,000 IU of vitamin D in patients with vitamin D deficiency did show a reduced risk of falls (RR, 0.91) [119].

Interestingly, a small 6 month study in patients over 70 years of age with a history of at least two falls in previous year and low 25(OH)D levels found that supplementation with a vitamin D dose of 800 IU/day for 6 months decreased self-reported fall number from an average of 3.76 ± 2.2 to 0.76 falls per year [120].

Concerning effects of active vitamin D analogs (calcitriol, alfacalcidol and eldecalcitol) on fall risk a very recent updated meta-analysis including 771 participants of published RCTs active vitamin D analogs reduced the fall risk by 19% [121].

## Hip protectors in the prevention of hip fractures

Diagnosis of osteosarcopenia has two important advantages. In fact, on one side it allows to identify a high-risk group that is at the same time prone to falls and have an increased bone fragility which in case of a fall may intrinsically increase the risk of hip fracture. On the other side, it gives to health practitioners a unique opportunity of active prevention which consists in supplementation of people with osteosarcopenia with adequate doses of vitamin D, if deficient, in order to improve muscle mass and function reducing both the fall risk and the propensity of bone to fracture after impact [33]. However, this form of prevention, although according to the majority of experts may statistically reduce the risk of fractures, clearly does not abolish it. Therefore, there is an urgent need for the synergistic use of a personal safety tool that may act downstream in the risk chain, attenuating the force transmitted to the femur during the fall. Therefore, osteosarcopenic patients at high risk of falls should wear a hip protector [122]. However, currently the use of these devices is limited by the significant physical restraint by them induced which paradoxically may even increase the incidence of falls and reduce compliance [123]. Therefore, in the next section, we will discuss state of the art and perspectives for application of hip protectors in clinical practice.

### Types of hip protectors

From the mechanical point of view, in the current market, hip protectors can be classified into passive and active safety systems. In passive hip protectors, the impact mitigation system is always operative, while in active hip protectors, it is triggered only when sensors embedded in the device detect an imminent fall [122].

In literature, passive hip protectors have been further distinguished into hard or soft, depending on the material choice and mechanism of protection. Passive hard hip protectors consist of plastic shells, made of a relatively stiff material, which form a bridge over the trochanter transmitting the impact force to the surrounding soft tissues [123, 124] (denominated “energy-shunting” mechanism) [125]. Instead, passive soft hip protectors usually exploit an “energy-absorbing” protection system, consisting primarily of elements characterized by high-deformability (foams or rubber), able to provide sufficient cushion to absorb most of the impact energy through elastic compressive deformation [126, 127]. Attempts have been made in creating a hybrid model, which combined the hard and soft-shell technologies [128].

One of the main common aspects of passive hip protectors available in the market is the necessity of specially designed underwear, with pockets in order to allow the correct placement of the pad over the region of the major trochanter. However, this feature has been identified as one of the main reasons for the low compliance to hip protectors, considering the difficulty associated with the actions of putting on and taking off them during bathing or other daily activities, for which the presence of a garment is seen by the patient as an obstacle [129, 130].

On the other hand, active hip protectors currently exploit “airbag” systems, similar to those present in the automotive field. Sensors monitor constantly the acceleration and position of the pelvis with respect to the ground and when the fall-detection algorithm predicts an imminent impact due to a fall, a trigger mechanism is activated to release gas, previously stored in a canister, which inflates the protective bag [131]. They are usually worn around the waist [132–134] for improved comfort and wearability.

### Clinical efficacy

The clinical efficacy of hip protectors in institutionalized subjects has been evaluated in three meta-analyses of RCTs [135–137]. Risk of hip fractures was found to be very variably but significantly reduced in all meta-analyses by the use of hip protectors in this setting. This reported quite high variability in the results (relative risk reduction ranging from 0.40 to 0.82) raised some degree of uncertainty around the benefit of using hip protectors in older institutionalized people. Moreover, these discrepant data somewhat attenuated the interest on the topic. In fact, over last 15 years, no further RCTs on hip protectors were undertaken. However, this inertia is also due to the intrinsic complexity of such clinical trials that, having hip fracture as endpoint, need very large sample size and long follow-up being consequently particularly expensive [138]. Moreover, several limitations of these studies have been pointed out by Robinovitch et al. [138]. First of all, their “intent-to-treat” approach aimed to assess the effect of assigning a treatment without investigating the effect of actually receiving the assigned therapy. As a result, the clinical efficacy of hip protectors was assessed comparing at the general level the reduction of risk of fracture between control and intervention groups. No information was given regarding whether the hip protectors were actually worn at the time of the fall by the single participants. Hence, the meta-analyses may suffer from an important bias given by the effective acceptance of the medical device by the participants. Moreover, in some cases [139, 140] the hip protectors used in the clinical trials were reported to have inadequate energy-absorption performance with respect to other devices available in the market in further systematic impact testing conducted by Laing et al. [124].

Randomized controlled trials conducted with “per-protocol” methods instead make up for the compliance bias measuring clinical efficacy by comparing outcome of falls when the hip protectors were worn with respect to unprotected individuals. In this regard, Kannus et al. [122] reported an 80% reduction of unadjusted hip fracture risk comparing falls vs without hip protectors. Interestingly, in three quarters of falls hip protectors were worn. Moreover, Bentzen et al. [141] reported a 64% decrease vs unprotected falls in hip fracture risk when the falling patients were wearing a soft hip protector, and a 59% decrease after falls with a hard-shell hip protector. Interestingly, about half of the falls occurred in patients wearing hip protectors.

Recently, promising results on the clinical effectiveness of hip protectors were obtained by Korall et al. [142] in a 12-month observational non randomized study. The study retrospectively reviewed fall incident reports of residents from 14 nursing homes in the Fraser Valley of British Columbia (Canada), assessing specifically if the hip protectors were actually worn at the time of falls. Importantly, an almost three fold reduction in hip fracture risk in patients wearing hip protectors when falls occurred was reported. Since patients were wearing hip protectors in 60% of falls, it was estimated that hip protectors could prevent yearly slightly more than 6 hip fractures per 1000 hospital beds. In addition, from the study reports positive compliance data could be inferred about tested hip protectors which were selected after thorough laboratory testing of their performance [124]. This underlines the importance of effective biomechanical modeling of hip protectors to be used clinically for the reduction of hip fracture risk.

Regarding the clinical efficacy of active airbag hip protectors, RCTs are lacking and current knowledge relies on a retrospective pilot study recently conducted in 11 residential care homes in the Netherlands [132]. The study compared at a population level hip fractures in the control and intervention periods before and after the introduction of the protectors in the protocol. Study results showed that hip and pelvic fractures occurred at a 45% reduced rate after use of hip protectors vs an almost stable (-12%) rate of falls. Furthermore, this result is probably an underestimation of the efficacy of the device, considering that only 45 hip airbags were introduced among > 900 individuals selecting patients at the highest risk of falling and that no fractures occurred in falls protected with airbag hip protectors. A randomized clinical trial is still needed to assess the real effectiveness of active airbag hip protectors.

### Biomechanical efficacy

In 2007 the IHPRG (International Hip Protector Research Group) was formed specifically to address the perceived barriers to clinical value of hip protectors. One of the main focuses was the formulation of uniform standards regarding testing methods for evaluating the biomechanical efficacy of hip protectors. In fact, without universally accepted systems able to assess their biomechanical efficacy, it would not be possible to preselect which type of hip protector may worth to be tested in clinical trials [143]. This system should reproduce realistically the response and the anatomy of the human body. Excessive compliance or stiffness would result in an incorrect evaluation of the reduction of peak force and of duration of the impact of the hip protector [144]. In 2009 an international consensus statement was released regarding recommendations for biomechanical testing of hip protectors [144]. In this document, it was suggested to use a test system consisting of a falling mass of 28 kg, impacting a pelvis surrogate with a compressive spring with stiffness of 47 kN/m at a velocity of 3.4 m/s.

The Canadian Standards Association (CSA) released in 2020 the regulation CSA Z325 [145], providing a reliable and effective method for mechanical testing the efficacy of these types of protective medical device.

Interestingly, the document does not identify specific criteria to approve or reject hip protectors, but rather recommends manufacturers to specify the protection level in the labelling of hip protectors, in order that physicians and patients can be aware of their choices. The concept may be similar to the star rating of EuroNCAP (European New Car Assessment Programme) in the automotive field, which provides consumer information on the safety of new cars, without being restrictive on their circulation, and which was positively correlated with overall improvement of vehicle safety [146]. However, this approach does not appear to entirely fit into the field of medical devices calling for a closer collaboration between engineers and biomechanics experts and clinicians in order to offer to patients only safe and effective products.

Two main experimental test campaigns were carried out investigating the performance of hip protectors in the market. The first one was conducted by Laing et al. in 2011 [125] and the second one by Keenan et al. in 2019 [147].

Keenan et al. [147] followed the indications of CSA EXP-08–17, an early version of the standard, released in 2017 which was published as an express document, subsequently withdrawn. A mass of 28 kg was released vertically at a speed of 3.2 m/s on hip protectors secured on an aluminum femur form, embedded in silicone soft tissue simulant. Importantly and pertinently to osteosarcopenia in humans, the characteristics of the muscles over the great trochanter have been shown to influence greatly the outcome of falls [126, 148–150]. A spring with a stiffness of 40 kN/m was attached between the drop weight and the impact plate, simulating the compliance of the human body, based on extensive tests conducted by Robinovitch et al. [151].

**Fig. 1:**
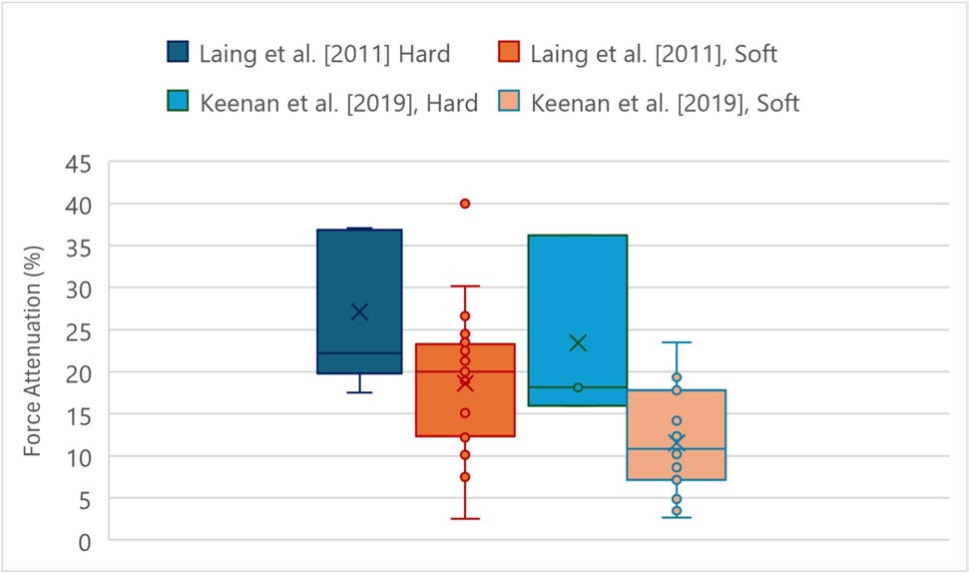
Analysis of force attenuation values of hip protectors in biomechanical testing campaigns of Laing et al. [121], and Keenan et al. [144], categorized by types of protector

As illustrated by Fig. 1, across both experimental campaigns the hard protectors performed better in terms of force attenuation with respect to the soft ones. Greater pad thickness and width and lower initial stiffness (measured at 500N of applied load) were positively associated with energy absorption during fall. In addition, hip protectors that bridged over the proximal femur were characterized by greater force attenuation values at low-to-moderate impact velocities likely due to their enhanced ability to shunt energy away from the proximal femur and into surrounding soft tissues. However, it was also found that for falls at higher velocities the benefit was almost canceled out with respect to devices which directly covered the proximal femur. In fact, over a certain threshold of impact severity, the protector was not able to dissipate all the energy of the impact away from the collision area, resulting in a phenomenon called ‘bottoming-out’. In fact, beyond this point, the devices come fully in contact with the hip region, but provide no additional energy absorption capabilities due to their high rigidity.

There is evidence of a third test campaign conducted by Holzer et al. [152], who tried to make up for the lack of any clear regulation at the time using the European Standard testing method for motorcyclists’ protective clothing (EN 1621–1) [153]. The results of the study were controversial. Soft hip protectors of the “energy-absorbing type” proved to have superior performance with respect to hard hip protectors of the “energy-shunting” type. However, this could be likely attributed, at least in part, to the test configuration, which consisted in a 5 kg steel mass impacting hip protector positioned on a 50 mm hemispherical steel anvil, with considerably smaller radius and surface area than the human hip. As a result, the hard hip protectors were not able to shunt the energy of the impact away from the hip region, on the surrounding tissues [154]. In addition, this testing method did not reproduce realistically the muscles overlying and surrounding the proximal femur [127].

Regarding the biomechanical testing of airbag hip protectors, Jeong et al. [155] performed experimental tests versus different types of passive hip protectors using a surrogate pelvis made of acrylonitrile butadiene styrene (ABS) of size determined by referring to the hip sizes of older women from South Korea. The effective impact force (force applied to the femoral area) in airbag hip protectors was found to be the second lowest among all hip protectors tested. Furthermore, the airbag managed to decrease the pressure applied on the hip by distributing the contact force of the impact on a larger area.

### Compliance

As already mentioned, the efficacy of the hip protectors is hindered by the adherence and acceptance of the patients.

A systematic Cochrane review conducted in 2002 by Van Schoor et al. [156] reported low to moderate primary acceptance, ranging from 37 to 72% and compliance between 20 and 92%, with median values of 68% (interquartile range 57–70%) and 56% (interquartile range 41–73%), respectively.

In general, soft hip protectors were found to be characterized by higher compliance with respect to hard hip protectors [157, 158].

Andrews identified six barriers to the hip protector acceptance and compliance [159]. The most common negative factor of hip protectors consisted in the discomfort associated to the tight-fitting garments, which are designed in order to place the hip protectors in the right position above the great trochanter, since this factor has been connected to efficacy [126]. The difficulty associated with putting on and taking off hip protectors was perceived as another strong obstacle to adherence, especially in individuals affected by upper and lower limb arthritis [160], due to the decrease of independence and autonomy, especially in using the toilet [130, 161]. Moreover, in one case report, a hip fracture was reported in a woman with Alzheimer’s disease who fell twice during one night while trying to go to the toilet [162].

A recent online survey conducted by Andrews [163] with members of the Royal Osteoporosis Society reported a major gap between the physical and psychological needs of people in wearing hip protectors. Users tend to put greater emphasis not only on comfort but also on the appearance of the protective garments, with respect to their performance. Respondents stated to prefer hip protectors discreet and hidden from view, reflecting a perceived feeling of embarrassment related to ageing and loss of independence of the older individual. It was reported that 65% of the over-70 subjects would prefer to wear a protector hidden under clothes, with respect to only 46% of under-60 subjects. Interestingly, the percentage of the interviewed people who would wear visible hip protectors, if they were appealing, was higher in younger subjects at risk (56% in under-60 subjects), with respect to an older population (33% in over-70). The findings were in agreement with previous studies showing that “compliers” were statistically younger than drop-outs [163]. The discreetness of the hip protector is a critical aspect in the hip protectors design, especially considering that the thickness of the pad has been associated with improved biomechanical performance [124, 144]. The study also underlined the difficulty to use hip protectors as a medical device in the context of medical care facilities [144].

The discomfort of wearing passive hip protectors is counteracted by the design of active hip protectors, which are usually worn like belts around the waist, since they inflate upon fall detection and do not need to be always positioned tightly over the hip region. Nemeth et al. [132] mentioned that all participants in their study accepted and tolerated well wearing the hip airbag during the intervention period. Some concerns still remain regarding the impossibility, as of today, of hiding these kind of devices under the clothes, due to the need of expansion of the cushion [159]. A pilot study conducted at Beaumont Hospital (Dublin, Ireland) is ongoing investigating the acceptability and effectiveness of two airbag devices available in Europe and CE marked, the completion of which is estimated in April of 2025 [164]. However, one detrimental aspect to the acceptance of airbag hip protectors is their affordability. Airbag hip protectors currently occupy a much higher-cost market segment with respect to the passive ones (up to 10 times) due to the presence of electronic components, sensors, and controllers. Hence, at the moment the distribution of these types of devices targets prevalently long-care environments and hospitals, rather than individual customers, given also the fact that, at the moment, they are not yet reimbursable by insurance or Medicare. Systematic review by De Bot et al. observed that cost-effectiveness of the hip protector is one of the key factors for the prevention of hip fractures in patients with higher fracture risk. [165].

Moreover, some studies suggested that the commitment of caregiving staff was a key facilitator of compliance [156, 160, 166]. However, due to the amount of continuative care required by residents and the workforce shortages, the overburdened staff often suggest other less demanding practices to reduce risk fracture in residential settings. In a recent cross-sectional study on staff’s insight into fall prevention solutions in long term care facilities [167], hip protectors resulted to be the third preferred method to prevent residents from experiencing fall-related injuries, after environmental design towards safety (clutter-free-environment and non-slip flooring) and protective equipment (crash mats and compliant flooring).

### New technologies

In order to overcome these limiting aspects, recently, innovative materials and concepts have been explored. One of the most promising strategies consists in the use of pads made of shear-thickening materials [168, 169]. These types of materials are characterized by an increase of viscosity with the rate of shear strain, such that they appear soft and comfortable while being worn and not stressed, and become stiffer, hence more resistant to impact, when they are subjected to a violent loading. Safety protective equipment exploiting this physical principle are widely spread in highly technical applications like the sport and motorbike fields, with promising results [170]. Hall et al. [169] conducted five consultation events involving older adults and care-sector staff in the Midlands and North West of England, as a feasibility study of Fall-Safe Assist hip protector, a new shear thickening product developed and patented by Hip Impact Protection. However, the compliance and effectiveness of these types of products on an older population for everyday use have not been yet verified with consistent clinical trials.

Another technological advancement which was suggested to improve the design of hip protectors is additive manufacturing, thanks to the possibility of adapting to the human body shapes. Yahaya et al. [171] published an optimization study on the printing parameters of additively manufactured hip protector pad in flexible thermoplastic polyurethane (TPU) by Fused deposition modelling, in order to maximize the force attenuation of the pad. Park et al. [172] developed impact-protection pants referring to the average size of older women in South Korea, with specific pockets for placing pads 3D printed in flexible TPU, with hexagonal mesh designed to improve energy absorption. In this case, the design and usability by elderly people were evaluated positively by both an expert and a subject group.

An alternative solution has been explored by Post et al. [173], with the development of a stick-on-hip protector, which adheres directly to the person’s skin, without the need for specific underwear. However, in this case, concerns have been reported among healthcare professionals on the possibility of skin irritation and limited adherence over prolonged usage.

### Numerical methods

The development of novel types of hip protectors could benefit from the use of innovative computational tools to reproduce the outcome of falls and provide insights into the level of protection provided. In particular, biofidelic numerical models of human anatomy aim at reproducing accurately organs and tissues in order to study their behaviour in critical conditions for which experimental studies are not feasible, like falls and accidents.

Fleps et al. conducted the first experiments with a surrogate of human lower extremities for the investigation of the effects of falls [174]. Skeletal hips and femurs from fresh frozen cadaver were used comprised of cartilage and ligaments. Ballistic gel was used to represent soft tissues, muscles and fat, showing comparable mechanical properties [175], with subject-specific moulds defining the geometries. Lower limb constructions composed of aluminum profiles and steel masses were used to guide the hip surrogate in a gravity-driven inverted pendulum and a ball-and-socket joint in correspondence with the feet to simulate a lateral fall.

The data acquired from load cells installed on the impacted surface and from high-speed recording were used to validate a Finite Element model (FEM) simulating the ex-vivo specimens and the fall conditions. A material mapping strategy previously developed by Enns-Bray et al. [176] was used together with material properties evaluated in previous studies [177–179]. Cartilage was modelled as a hyperelastic material without viscoelastic effects [180, 181]. The results predicted by the numerical simulations were highly correlated with the result of ex-vivo experiments [182] in terms of peak impact force and effective pelvic stiffness, with a mean square error of less than 15%. The assessed numerical model was also used to investigate the influence of hip protectors on fracture risk [183], finding that 7 out of 16 femoral fractures were successfully avoided out of the 56 simulated impact conditions thanks to the use of a foam-based soft-shell hip protector, with stiffness and energy absorption values in the range of previous literature studies [124]. The efficacy in reducing fracture risk was found in range of previous trials; however, it was reported that in severe falls, still in the range of observed impact velocities, the force attenuation of hip protector was not sufficient to prevent fractures. Moreover, it was suggested that for subjects characterized by high BMI the hip protector could slightly elevate hip fracture risk by acting as a force concentrator.

The limitations of the work consisted in the lack of real human muscles, fat and skin in the lower-extremities surrogate specimen and the effect of muscle activation. In this context, Kim et al. [184] recently conducted experiments with participants instructed to relax or contract hip muscles and found that the stiffness and energy absorption were increased up to 59% with muscle activation.

### Market analysis

Recent market analysis from Verified Market Research [185] identified an actual market of about 50 million dollars for hip protectors, with expected growth up to 74 million dollars for year 2028, with regions of highest growth potential being Pacific Asia and North America.

## Hip fracture prevention: a combined strategy with vitamin D and hip protectors

Hip fractures are among the most clinically relevant causes of elevated morbidity and mortality in the older people, and are already associated with a huge economic, social and health burden which is expected to become even higher as a consequence of progressively increasing life expectancy and prevalence of older people. Thus, prevention of hip fractures already represents and will become an even greater challenge for global public health in the near future.

**Fig. 2:**
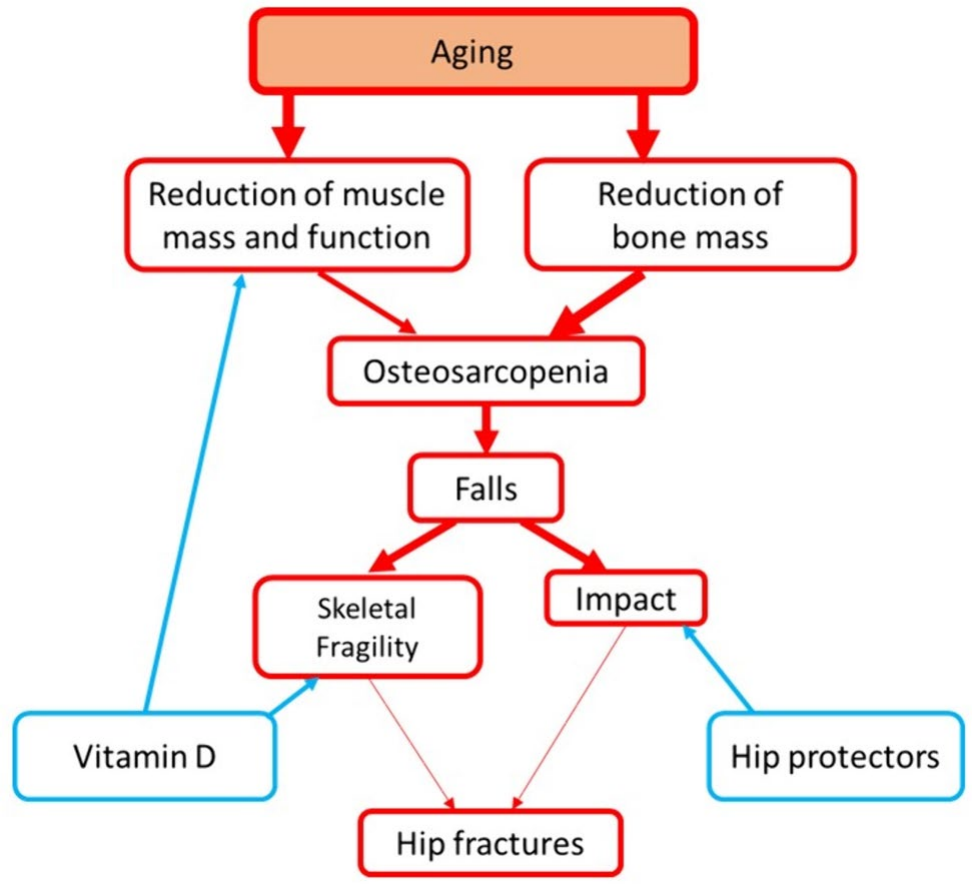
Pathophysiology of hip fractures in the elderly and strategies for prevention. Reduced thickness of the arrows indicates attenuating effect of preventive measures. Arrows in blue indicate prevention strategies

Osteosarcopenia is an emerging clinical condition which is highly prevalent in the older people and predisposes to hip fractures increasing propensity to falls through decreased muscle mass and function. In this regard, vitamin D has been proposed to act through one of its major extraskeletal actions, i.e. improving muscle structure and performance. Moreover, osteosarcopenia incrases fracture risk also decreasing the resistance of bone to impact and vitamin D may positively act also at this level through its classical pro-bone mineralization action (Fig. 2). However, type and severity of impact of bone after falls may per se constitute a major risk of fracture in osteosarcopenic subject for those in whom falls are not prevented by vitamin D (or other pharmacologic preventive measures) or in whom skeletal fragility persists despite vitamin D. Therefore, there is a third downstream possible preventive measure which is represented by hip protectors which physically attenuate the impact of bone after fall (Fig. 2).

**Fig. 3:**
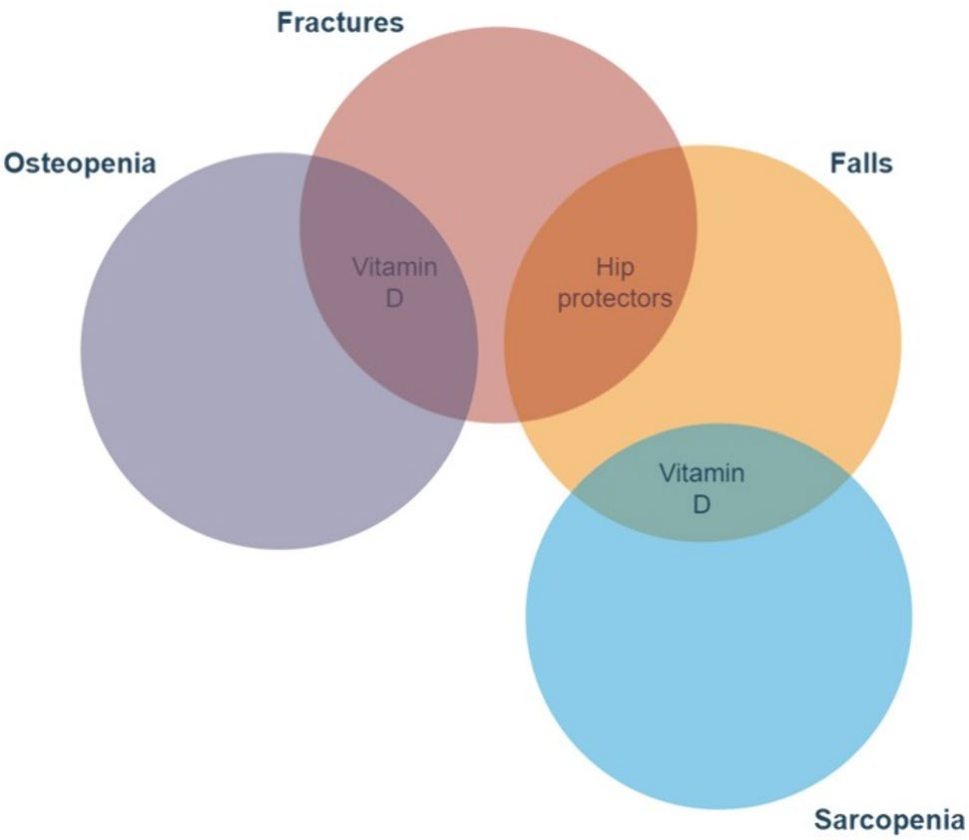
Visual representation of synergistic effect oh Vitamin D and Hip protectors on Risk of Fractures in Osteosarcopenia

**Fig. 4:**
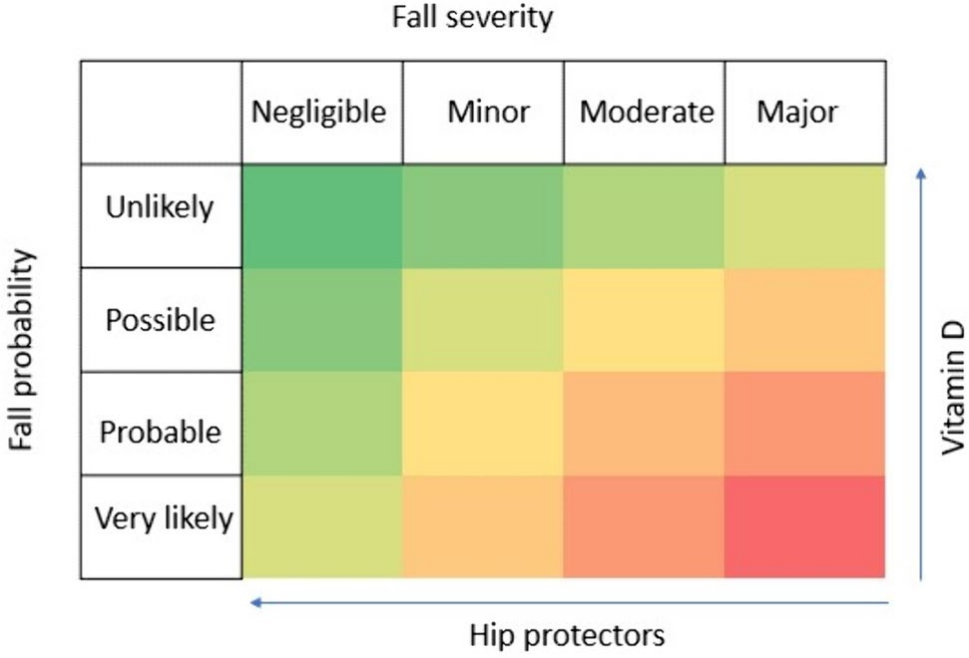
Risk matrix diagram relative to the effects of hip protectors and Vitamin D on falls. Green = lowest risk. Red = highest risk

In fact, the combined use of vitamin D and hip protectors synergistically taking advantage of active and passive prevention could be an innovative low-cost strategy for preventing hip fractures acting at different levels of the risk chain (Fig. 3) producing a theoretical continuum from high to low risk of fractures by reducing the likelihood to fall and the negative effect of impact on bone (Fig. 4). Practical challenges of implementing such strategy in clinical settings may be represented by patient compliance, cost, and accessibility. In fact, combining two different preventive approaches could add to patient burden in terms of compiance and may not be well accepted, particularly by older patients. Moreover, it can increase the cost for the patients unless found so effective in fracture prevention to be reimbursed at least in some Health systems. Finally, in some Countries, such as Italy there are still important limitations in the reimbursement of vitamin D [186] and this may cause a reduced access to its use.

 In our review, we pointed out that the proposed strategy may have drawbacks besides the variability that can be found in the literature on entity and consistency of protection conferred by either vitamin D and hip protectors. First, there are no clinical studies supporting this strategy which therefore needs to be proven effective in clinical trials despite the attractive and sound pathophysiological basis of the concept. Second, other important factors potentially conditioning the combined strategy such as compliance, costs and availability may currently represent drawbacks of this approach (Table 1). Nevertheless, the development of different forms of cholecalciferol which may increase the long-term compliance to the treatment [90] and the development of more user-friendly and effective protectors also through innovative materials [187] may be a promising basis for future studies in which the synergistic mechanism of the two measures should be tested. In order to ensure replicability and validate the results, such studies should follow clear methodological guidelines. In fact, it can be suggested that accurate definition of osteosarcopenia should be used, whereas subjects with secondary causes increasing the risk of falls such as use of drugs and cognitive impairment should be excluded. Moreover, use of innovative hip protectors preselected based on adequate biomechanical tests and accurate methods of recording fall episodes shoud be used. Finally, four arms of the study, which therefore should enroll a large population with sufficiently long follow-up, could be proposed randomly attributing osteosrcopenic subjects with hypovitaminosis D at study entry to either no treatment vs vitamin D or hip protector alone vs vitamin D combined with hip protectors.

**Table 1:** Qualitative comparative assessment of vitamin D and hip protectors on different clinical outcomes

Hip fractures risk reduction strategies		Risk reduction mechanism	Compliance	Cost	Effectiveness	Access
Hip protectors	Hard	Passive	–-	+	+ +	+
	Soft	Passive	–	+	+	+ +
	Airbag	Active	-	–-	+ + +	-
Vitamin D		Active	+ + +	+	+ +	+ + +

## Conclusion

Our review focused on pathophysiology of hip fractures and on their two main underlying mechanisms, osteosarcopenia and falls. Moreover, we also reviewed two possible approaches to hip fracture prevention such as vitamin D and hip protectors. Finally, due to their potentially synergistic mechanism of action we illustrated the possible advantages and drawbacks of a combined approach in clinical practice. Due to relatively easy and inexpensive approach to a huge health, social and economic problem we conclude that this combined approach is worth to be explored in clinical studies. Future research particularly devoted to the implementation of more user friendly and effective hip protectors may further support this combined approach in the future.

## Data Availability

No datasets were generated or analysed during the current study.
